# Anti-cancer mechanisms of natural isoflavones against melanoma

**DOI:** 10.1016/j.heliyon.2024.e28616

**Published:** 2024-03-27

**Authors:** Cheng Liang, Ping Wang, Mengzhen Li, Rong Li, Keng Po Lai, Jian Chen

**Affiliations:** a Guangxi Key Laboratory of Tumor Immunology and Microenvironmental Regulation, Guilin Medical University, Guilin, China; b Key Laboratory of Environmental Pollution and Integrative Omics, Guilin Medical University, Education Department of Guangxi Zhuang Autonomous Region, Guilin, China

**Keywords:** Isoflavones, Melanoma, Molecular mechanisms, Proliferation, Invasion

## Abstract

The incidence of skin-related neoplasms has generally increased in recent years. Melanoma arises from malignant mutations in melanocytes in the basal layer of the epidermis and is a fatal skin cancer that seriously threatens human health. Isoflavones are polyphenolic compounds widely present in legumes and have drawn scientists’ attention, because they have good efficacy against a variety of cancers, including melanoma, without significant toxic side effects and resistance. In this review article, we summarize the research progress of isoflavones in melanoma, including anti-melanoma roles and mechanisms of isoflavones via inhibition of tyrosinase activity, melanogenesis, melanoma cell growth, invasion of melanoma cells, and induction of apoptosis in melanoma cells. This information is important for the prevention, clinical treatment, and prognosis and survival of melanoma.

## 1 Introduction

Melanoma is a malignant tumor that occurs when melanocytes mutate, and its incidence has been increasing worldwide in recent years [1]. Melanoma accounts for the majority (∼73 %) of skin cancer-related deaths [2]. Melanoma mainly occurs in the skin, but also in normal non-cutaneous sites, such as the eyes, digestive system, genitourinary system, and nasopharynx [1]. Melanoma has an low responsiveness, and resists existing targeted treatments, severely hampering its treatment and control. Because existing melanoma treatments are inadequate, new melanoma treatment options are urgently needed. Natural isoflavones were reported to have anti-tumor potential. In this article, we will review the literatures on the anti-melanoma roles of isoflavones. Also, we will summarize it anti-melanoma mechanisms through inhibiting tyrosinase activity, reducing cell growth and invasiveness of melanoma, and inducing apoptosis of melanoma cells.

## 2 Epidemiology of melanoma and limitations of current melanoma therapies

Compared to other tumors, the incidence of cutaneous melanoma (CM) is increasing significantly faster [3]. CM is an aggressive form of skin cancer that originates in the melanocytes. According to the data from American Cancer Society, it is estimated that there were 100,640 new cases and 8290 deaths from melanoma in the United States in 2024 [4]. Melanoma can be caused by many factors, its main risk factors include gender, age, sun exposure, ultraviolet radiation (UVR), number of moles, and presence of family history of melanoma [5]. The impact of melanoma on gender is age-related, with females being more frequently affected in younger age groups, whereas males predominate over 55 years of age when sex-related incidence data are analyzed [6]. The incidence increases with age, with more new cases occurring between 65 and 74 years of age, and the median age of onset being 64 years [7]. Approximately 60%–70% of melanomas caused by UVR [8]. Genetic alterations due to intense UVR, consisting of DNA damage, genetic mutations, reactive oxygen species (ROS) accumulation, oxidative stress, and inflammatory responses such as infiltration of macrophages and neutrophils, all of which stem from the malignant transformation of melanocytes [[9], [10], [11], [12]]. Melanomas are prone to mutations and have the highest mutation frequency in all cancers, with a maximum of 100 mutations per Mb [13]. BRAF mutations occur in 60% of melanomas and NRAS mutations occur in 15%–20% of melanomas [14]. Mutations in the BRAF gene may not directly cause melanoma, but the risk of melanoma increases with the addition of environmental factors. Mole production is a precursor lesion leading to melanoma or a high-risk marker for melanoma development. About 25% of melanomas develop from a pre-existing mole [6]. Almost 80% of melanoma patients report changes in preexisting nevi [15]. The overall number of moles in an individual has the greatest impact on the risk of developing melanoma. Normally, the number of atypical moles represents the risk of developing melanoma, that is, the higher the number, the greater the risk. One of the most important risk factors for melanoma is family history. In families with a genetic predisposition, there is a 5%–10% chance of developing melanoma. Different locations can also influence the development of melanoma, which occurs mainly in areas exposed to intermittent intense sunlight, including male back and female limbs [15]. Major risk factors for developing melanoma also include early intermittent high-intensity sun exposure.

In addition, melanoma can cause serious complications because it usually metastasizes early, and the median survival for patients with metastatic melanoma is 5–8 months [16]. Because melanoma tends to metastasize and is clearly resistant to chemotherapy, it is often not treatable. This results in a trend towards a poorer prognosis for melanoma. The 5-year survival rates for cutaneous melanoma were 97% (Phase IA), 84% (Phase IB), 68% (Phase II), 55% (Phase III), and 17% (Phase IV) [17]. This prognosis worsens if melanoma happens in mucosal areas [18].

Current treatment for melanoma included surgical resection, chemotherapy, targeted therapy, and immunotherapy. Surgical resection of the tumor is still the most important treatment for melanoma, but for metastatic melanoma, surgical treatment is often insufficient, and needed to be followed by drug chemotherapy. The FDA-approved first chemotherapeutic agent for melanoma is dacarbazine, which has a median survival of 5–11 months and a 1-year survival rate of only 27% [[19], [20], [21], [22]], but the side effects of chemotherapeutic agents remain inevitable. Targeted therapies and immunotherapies are therefore increasingly used. Immune checkpoint inhibitors are one of the most effective treatments for metastatic melanomas [23]. Melanomas can manipulate immune checkpoints through PD1 [23], PD-L1/2 [22], and CTLA-4 [24]. Despite these breakthroughs in cancer treatment, a large proportion of patients still do not respond well to these drugs, and some responsive patients develop secondary resistance [25]. More importantly, the treatment is expensive, and the side effects can be serious if they occur. Therefore, a novel drug is urgently needed for the treatment of melanoma. Isoflavones could be one of the good choices, because of their various biological effects.

## 3 Source, structure and clinical use of isoflavones

Isoflavones are phenolic compounds formed during plant phenylalanine metabolism by cyclization of cinnamoyl-CoA following side chain extension based on the phenytoin ring. Their 3-phenyl derivatives are isoflavones. Isoflavones are secondary metabolites in plants and their chemical formula is C_15_H_10_O_2_ (Table 1). The basic backbone of isoflavones is 3-phenylchromene dihydropyran. They are isomers of aromatic oxygenated heterocycles and flavonoids that are constitutionally related to endogenous estrogen 17-β-estradiol (E2), usually in a conjugated form, and have a molecular weight similar to E2 (222.24 g/mol). Isoflavones contain two benzene rings (A and B) that are connected by a heterocyclic pyran residues. Phenyl ring B was found to be opposite to the heterocyclic pyran residue and attached to carbon 2 of heterocyclic pyran in all flavonoids except isoflavones. This structural difference clearly differentiates isoflavones from flavonoids [26]. Isoflavones exist in two forms: glucoside and glycoside [27,28] and are assimilated in the small and large bowels of humans [29]. The glycosidic form of isoflavones is absorbed by the body faster than glucosides and is hydrolyzed in the proximal intestine to glycosidic aldehydes, which are biologically active and better absorbed [30,31]. Isoflavones are present in human blood mainly as glucuronides (75%), sulfates (24%), and aglycones (<1%) [32].

**Table 1:** Physicochemical properties of isoflavones.

Isoflavone	Phytoestrogen
Category	Polyphenolic substance
Chemical name	3-phenyl-4H-1-benzofuran-4-one
Sensitivity	Sheet or needle
Density	1.239g/cm3
Molecular formula	C_15_H_10_O_2_
Molecular weight	222.24 g/mol
Boiling point	367°Cat760 mmHg
Chemical Structure	
Main source	Beans
Common isoflavones	Daidzein、formononetin、genistein

Isoflavones are rich in our daily food products. The major nutritional source of isoflavones is legumes [33]. Soybean isoflavones are present at approximately 1.5 mg/g and include genistein and daidzein [27]. Other dietary sources of isoflavones are chickpeas as well as other legumes and plant products such as fruits, vegetables, and nuts, which also contain small amounts of isoflavones [34]. Soy consumption has been shown to significantly improve human health [35] and increased soy intake reduces cancer risk [36]. It has been found that isoflavones in soybeans have many physiological and pharmacological functions, so they have become a research hotspot.

Isoflavones have become one of the important nature compounds in the field of life sciences. Nowadays, most of the isoflavone-related drugs are used as health products in the market. For example, soybean isoflavone tablet is a functional health food made from natural red yeast rice, soy isoflavone and Ganoderma lucidum extract [37]. It has the functions of delaying aging, lowering cholesterol, reducing the occurrence of cardiovascular disease, and improving osteoporosis in women. Kudzuvine isoflavone capsule is made from Kudzuvine isoflavone, biocalcin, zinc lactate and vitamin D as the main raw materials. It has health care effects such as delaying aging, cosmetic breast enlargement, eliminating menopause, increasing bone mineral density, and preventing cardiovascular and cerebrovascular diseases. Probiotic soy isoflavones are soft capsule products, which contains daidzein and genistein. It can reduce hot flashes, maintain youthful vitality, reduce gynecological inflammation, eliminate menopausal syndrome, and increase estrogen levels [38].

Due to the structural similarities between isoflavones and estrogens, they can bind to the estrogen receptor [39]. Thus, isoflavones may act as antagonists or agonists of endogenous estrogens [40]. Potential phytoestrogenic effects of isoflavones are cell type specific and depend on their ability to bind to the alpha or beta estrogen receptors. Isoflavones inhibit cell proliferation and stimulate apoptosis [41], and their antioxidant effects in the proliferation and differentiation of malignant cells enable them to be prospective anticancer compounds. Breast cancer is one of the most frequent terminal cancer in women [42] and studies have found that breast cancer incidence is lower in Asia than in Western countries. In Asian populations, the diet is dominated by soy products, in which individual's isoflavone intake can be as high as 50 mg/day. In comparison, the mean intake of isoflavones is less than 2 mg in Western countries, but may be higher in menopausal women [43]. In addition, some studies suggested that high intake of isoflavones in childhood or maturity may reduce the risk of breast cancer in later life [44]. Shu X.O. et al. demonstrated that consumption of soy isoflavone-rich foods reduced the risk of death by 29% and cancer recurrence by 32% in women who diagnosed with breast cancer [45]. According to the study conducted by Islam M.A. et al., the effect of isoflavones on inhibition or activation of cell proliferation depends on the specific ratio between estrogen receptor alphaα and estrogen receptor beta in cells [46].

Also, isoflavones inhibit inflammation, inhibit fatty acid synthesis [47], promote lipid synthesis and accumulation, and improve lipid metabolism [[47], [48], [49]]. Isoflavones ameliorated oxidative stress-induced endothelial nitric oxide synthase (eNOS) uncoupling and inhibited oxidative LDL-induced vascular inflammation [50]. In vascular endothelial cells, isoflavones enhance eNOS activity and inhibit ROS production [51,52]. Owing to the potential phytoestrogenic effects as well as anticancer and antioxidant activities of isoflavones, in-depth studies of their anti-melanoma mechanisms have revealed that isoflavones exert their anticancer effects through multiple targets and signaling pathways. This paper will discuss the use of isoflavones in the clinical management of melanoma.

## 4 Anti-melanoma pharmacology and mechanism of action

### 4.1 Mechanisms of melanin production

**Fig. 1:**
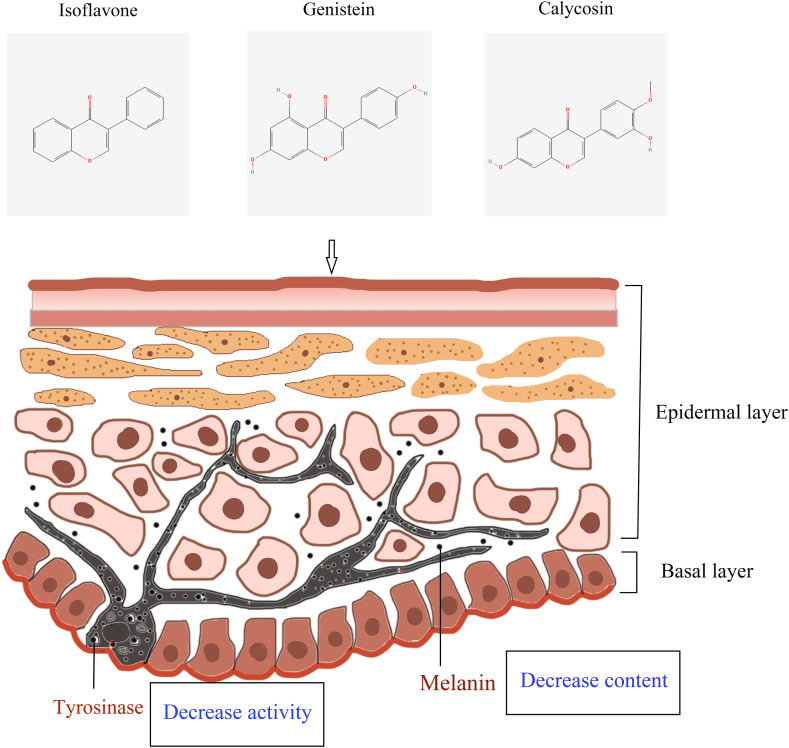
**Isoflavones reduce melanogenesis.** Isoflavones such as genistein and calycosin reduce melanogenesis by decreasing tyrosinase activity and reducing in melanin content.

**Fig. 2:**
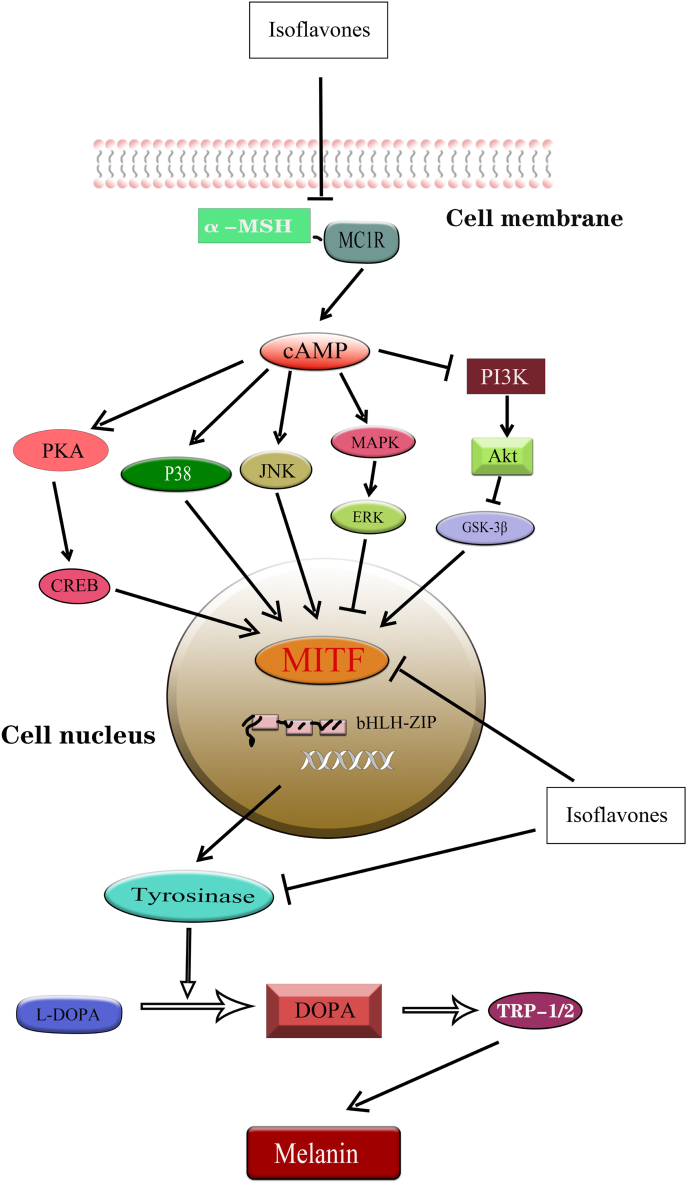
**Anti-melanoma roles of isoflavones through the regulation of different signaling pathways.** By inhibiting the binding of α-MSH to its receptor MC1R, isoflavones attenuate the activation of three major MAKP signaling pathways including p38, JNK, and ERK signaling, increase reactive oxygen species, promote tumor cell apoptosis, and reduce the activity of tyrosinase, thereby reducing the production of melanin and achieving anti-tumor effects on melanoma.

Overaccumulation of melanin or overexpression of tyrosinase (TYR) can lead to a variety of skin disorders, including wrinkles, aging skin spots, melasma, freckles, moles, and, in severe cases, melanoma [53]. TYR, a multifunctional copper-containing oxidase, is a key enzyme concerned with melanin production [54] (Fig. 1). TYR and TYR-associated proteins (TRP-1 and TRP-2) are transported early from the nucleus to melanosomes and catalyze melanin synthesis. TYR is able to catalyze the oxidation of l-tyrosine or L-3,4-dihydroxyphenylalanine (l-DOPA) to DOPA quinone, which is a key component of melanin synthesis [55](Fig. 2). It has been found that α-melanocyte stimulating hormone (α- MSH) binds to melanocortin 1 receptor (MC1R) to activate adenylate cyclase activity during melanin synthesis, which triggers intracellular cAMP production, and subsequently activates response element-binding protein (PKA/CREB) and three major mitogen-activated protein kinase (MAPK) family: JNK, MAPK/ERK, and p38 MAPK [56]. Activation of PKA/CREB, JNK, and p38 MAPK pathways elicits expression of basic helix-loop-helix leucine zipper (bHLH-ZIP), microphthalmia-associated transcription factor (MITF), TYR, and their associated protein TRP to promote melanogenesis [57].

### 4.2 Inhibition of tyrosinase activity and reduction of melanogenesis by isoflavones

Isoflavones act as chelating agents and are able to bring copper ions to the active site of tyrosinase with the tyrosinase-specific polyhydroxyphenolic structure. The high isoflavone-Sappanone A, isolated from hematoxylin, can inhibit tyrosinase activity and lead to a decrease in melanin production by suppressing the expression of tyrosinase gene in mouse B16 melanoma cells in a dose-dependent manner [58]. NBI (4′, 7-dihydroxy-3' - (3-methyl-2-butenyl)), an isoflavone isolated from Serratia species, also inhibits tyrosinase activity in a dose-dependent manner, leading to reduced melanin levels [59]. Also, NBI activated GSK3β and ERK (phosphorylation) that inhibit melanogenesis in B16 melanoma cells. And, the use of Akt/GSK-3β and MAPK/ERK specific inhibitors could reverse the NBI reduced melanin's level, suggesting negative regulation of these pathways in melanin production. In addition, activation of the PI3K/Akt pathway was reported to promote melanoma cell survival and proliferation. Also, Akt activation result in inhibition of cell apoptosis, thereby increasing the viability of melanoma cells [60]. In addition, PI3K/Akt pathway can enhance the migration and invasion of melanoma cells via the regulation of signaling pathways associated with cytoskeleton and cell-cell adhesion [61]. Aberrant activation of the PI3K/Akt pathway has also been implicated in melanoma cell resistance to anticancer therapies [62]. Activation of Akt can lead to the development of multiple resistance mechanisms through the regulation of cell cycle, DNA repair and apoptotic escape, making melanoma cells more resistant to chemotherapeutic drugs and targeted therapy [63].

### 4.3 Isoflavones reduced melanogenesis through regulation MITF

As mentioned above, MITF is a key mediator of isoflavones-reduced melanogenesis. MITF, a class E basic helix-loop-helix protein 32, is important in melanogenesis and proliferation of melanocyte [64]. MITF gene is reported to be amplified in about 20% of melanoma [65], so targeting MITF could be a promising approach for treating melanoma. Cajanin, an isoflavone isolated from yellow sandalwood florets, downregulates mRNA and MITF protein levels, resulting in dysregulation of CREB and ERK signaling, and decrease in melanin synthase (including TYR, TRP-1, and TRP-2) and melanin content [66]. Similarly, it was found that *o*-dihydroxyisoflavone from fermented soybeans in Korea also reduced melanin synthesis in B16 melanoma cells, comparable to that following treatment with kojic acid, a validated whitening agent [67]. RT-PCR results indicated that depigmentation due to hydroxyisoflavone-induced transcriptional repression of several melanogenic genes including MITF. Immunoblotting confirmed that inhibition of MITF expression caused a decrease in the expression of TYR, TRP-1, and TRP-2. Calycosin is one of the most potentially bioactive isoflavonoids in Astragalus membranaceus. Calycosin significantly inhibited the expression of melanin-related genes (including MITF, TYR, and TRP-1) in B2F16 melanoma cells to regulate MSH-induced melanogenesis. Further studies revealed that calycosin-inhibited of melanogenesis by regulating PKA/CREB and p38 signaling pathways [68]. Downregulation of MITF expression by activated Akt/GSK-3β signaling decreases transcription of TYR and TRP-1 [69]. Similarly, activation of the MAPK/ERK pathway can affect MITF activity and stability through phosphorylation, leading to reduced melanin synthesis [70]. Thus, PKA/CREB and MAPK signaling pathways may be new therapeutic targets for anti-melanoma drugs.

#### 4.3.1 Inhibition of melanoma cell growth

Russo et al. found that genistein protected DNA, suppressed UVR-induced oxidative DNA destruction, and exhibited superoxide dismutase-like effects, leading to inhibit the development of malignant melanoma [71]. In the study of melanoma cell, Yan et al. found that genistein decreased the phosphorylation of melanoma cytoskeleton-associated proteins, increased p53 content, and decreased transcription factor c-Myc, thereby repressing the growth and guiding the differentiation of B16 melanoma cells [72]. The in vivo experiments by Record IR also demonstrated that feeding of genistein could inhibited solid tumor growth by 16% in the mice inoculating with B16 melanoma cells [73]. In which, the plasma genistein concentration in the mice was 1.1 μM, which is similar to the reported level of genistein in human with daily soybeans or soybean products consumption. A similar effect on the tumor suppression was observed in another study that genistein could mediate melanoma growth by downregulating EP3 receptor and decreasing IL-8 expression, leading to inhibit melanoma cell proliferation in oral, uveal and skin [74].

#### 4.3.2 Inhibition of migration and invasion of melanoma cells

The migration and invasion abilities are important to the metastasis of melanoma through the regulation of several key factors and mechanisms. Protein tyrosine phosphorylation underlies the interaction of melanoma cells with the extracellular matrix (ECM). Yan et al. found genistein inhibited tyrosine phosphorylation at the cell periphery when BL6 melanoma cells adhere to each other and interact with the ECM [75]. The ability of BL6 cells to invade the basement membrane was significantly reduced when accompanied by adhesion-induced inhibition of protein tyrosine phosphorylation. Inhibition of adhesion-induced protein tyrosine phosphorylation and disruption of cell-ECM interactions can effectively influence the invasive cascade of malignant tumor cells. Therefore, genistein can be used for treatment against malignant tumor metastasis.

Isoflavones also play a significant role in angiogenesis, Farina et al. found that intraperitoneal injection of 10 mg/kg/day of genistein reduced tumor-induced angiogenesis in mice implanted with melanoma cells, and similar anti-angiogenic effects were observed in mice fed with soybeans [76]. Vasculogenic mimicry (VM) is a novel pattern of tumor microcirculation formed by invasive melanoma cells. Genistein represses VM of uveal melanoma cells both in vivo and in vitro [77]. VM can be adjusted by modifying the performance of endothelial- and epithelial-specific genes, including vascular endothelial (VE)-cadherin. In contrast, genistein decreased VE-cadherin expression, thereby inhibiting angiogenesis.

A study on the effect of dietary isoflavones on melanoma lung metastasis, mice were fed a basal diet or supplemented with the isoflavones genistein and daidzein before intravenous injection of melanoma cells [78]. Tissue samples were obtained two weeks later to measure the number and size of lung tumors. The supplement of isoflavones genistein and daidzein significantly reduced the tumor size and number as compared to control group, suggesting that isoflavones have anti-angiogenic effects and play an essential function in the countering of metastasis of malignant tumors [79].

#### 4.3.3 Effect on cell cycle progression in melanoma

Isoflavones are reported to control the cell cycle and proliferation of melanoma via targeting key regulators of cell cycle. It was found that the introduction of genistein caused melanoma cells arrest in G2 phase through the impairment of CDK1 dephosphorylation, but not that of CDK2 [80]. But another study using mouse B16–F1 melanoma cells showed that genistein prevented G1 to S phase transition via the suppression of cyclin E/CDK2 activity and induction of p21(Cip1/WAF1) expression [81]. In an ocular malignant melanoma study, mice were subjected to treatment with 25, 50 and 100 μM genistein. Immunofluorescence analysis of cyclin D1 was then performed using confocal laser scanning microscopy, the outcome demonstrated that the expression of cell cycle protein D1 was increased in the groups given 25 μM and 50 μM of genistein [82]. In contrast, administration of 100 μM genistein significantly decreased cyclin D1 expression. This reveals a biphasic influence of genistein on the expression of cyclin D1 in malignant melanoma cells. In addition, Wall et al. showed that fibrillar collagen (FC) mediates cell cycle arrest via the tyrosine kinase receptor DDR2 [83]. Genistein, as a broad-spectrum tyrosine kinase inhibitor, inhibited FC-induced cell cycle arrest.

#### 4.3.4 Induction of apoptosis in melanoma cells

Isoflavones are found to target many regulators of immune system and apoptotic factors of melanoma. Guo et al. found that isoflavones interfere with inflammation and affect melanoma [84]. In evaluating the effects of genistein on the immune system of B6C3F1 mice, it was observed that cytotoxic T-cell activity in mice treated with genistein was enhanced with increasing dose, and significant changes could be observed, especially at medium and high doses. In addition, interleukin-2-stimulated natural killer (NK) cell activity in B16F10 tumor model was considerably increased and basal splenocyte proliferation was enhanced in the treatment of genistein xanthine, suggesting that dynamin flavonoids enhance host resistance.

Kluger found that the novel isoflavone derivative phenoxodiol induced apoptosis by restraining the expression of X-linked inhibitor of apoptosis protein (XIAP) in melanoma cells, and melanoma YUMAC cells exposed to phenoxodil for 4 h displayed a decrease in the level of XIAP [85]. In addition, changes in XIAP expression were found to correspond to increased caspase-3, -8, and -9 activities. Rigano et al. found that isoflavone-rich natural products increased ROS production [86]. The canonicity of isoflavones facilitate ROS generation or trigger cellular oxidative stress may be a central decisive factor in the induction of apoptosis by phenolic compounds. Excess ROS activates cytoplasmic protein kinases and MAPK cascades, and also leads to mitochondrial damage, loss of membrane potential and cytochrome *c* release, which leads to apoptosis. Endoplasmic reticulum (ER) maintains intracellular protein homeostasis by regulating protein synthesis and translocation. Endoplasmic reticulum stress-induced apoptotic pathways consist of activation of the transcription factors C/EBP and homologous protein (CHOP) and suppression of the anti-apoptotic factor Bcl-2. A375 cells were treated with phytochemicals, such as Kaempferol, Genistein, and 3,3 ′-diindolylmethane, to detect ROS-mediated p38 MAPK and p53, and ER stress-mediated proteins in the mitochondrial apoptotic pathway [87]. It was found that the expression of phospho-p38 and p53, phospho-eIF2a, BAX, and CHOP were increased, but decreased in Bcl-2. This result suggested that isoflavones were efficacious in attracting apoptosis in melanoma cells.

## 5 Conclusions and perspectives

Isoflavones can inhibit the activity of melanoma cells through multiple targets and mechanisms, indicating that isoflavones can be used as new candidates for anti-melanoma drugs. Presently, most studies have concentrated on the in vitro effects of isoflavones on melanoma, and more in vivo and clinical studies are desirable to fully assess the efficacy and safety of isoflavones for treating melanoma. Because isoflavones are natural compounds, the relationship between their concentration and anticancer efficacy is not a simple linear relationship, and the specific effects depend on the type of isoflavones, mode of use, and individual differences of patients. So, it is necessary to determine the optimal concentration of these compounds for clinical treatment of melanoma. And, some isoflavones have even been found to reverse drug resistance in tumor cells. Therefore, isoflavones can be used as part of a comprehensive cancer treatment regimen, in combination with other chemotherapeutic drugs, radiotherapy or immunotherapy, which may produce synergistic effects and improve the efficiency of treatment. Although the anticancer potential of isoflavones is remarkable, further studies are still needed to explore their mechanism of action, optimize treatment regimens, and determine specific therapeutic effects. In addition, researchers are exploring and improving the bioavailability, stability and toxic side effects of isoflavones.

## Funding statement

This research is supported by the Guangxi Natural Science Founation (No. 2020GXNSFAA297057), Guangxi Science and Technology Base and Talent Special Project (No. AD23026092).

## Data availability statement

No data was used for the research described in the article.

## CRediT authorship contribution statement

**Cheng Liang:** Visualization, Validation, Software, Resources, Methodology, Formal analysis, Data curation, Conceptualization. **Ping Wang:** Visualization, Validation, Software, Resources, Methodology, Formal analysis, Data curation. **Mengzhen Li:** Validation, Software, Resources, Methodology, Formal analysis, Data curation. **Rong Li:** Writing – original draft, Validation, Software, Resources, Methodology, Investigation, Formal analysis, Data curation, Conceptualization. **Keng Po Lai:** Writing – original draft, Validation, Supervision, Resources, Project administration, Investigation, Formal analysis, Data curation, Conceptualization. **Jian Chen:** Writing – original draft, Supervision, Project administration, Investigation, Formal analysis, Data curation, Conceptualization.

## Declaration of competing interest

The authors declare that they have no known competing financial interests or personal relationships that could have appeared to influence the work reported in this paper.
